# Reviewed article: Silva PT, Toledo LV, Freitas BAC, Feitosa VA. Epidemiological profile, completeness of notifications, and factors associated with the adequacy of treatment for syphilis during pregnancy: a cross-sectional study, Ubá, 2018-2023. Epidemiol Serv Saude. 2026:35;e20250056

**DOI:** 10.1590/S2237-96222026v35e20250056.b

**Published:** 2026-03-20

**Authors:** Lucas Vinícius de Lima

**Affiliations:** 1 Universidade Estadual de Maringá, Maringá, PR, Brazil

## Peer review B

## Questionnaire

Does the manuscript contain new and significant information to justify publication? Yes

Does the Abstract (Summary) clearly and accurately describe the content of the article? Yes

Is the problem significant and concisely stated? No

Are the methods described comprehensively? Yes

Are the interpretations and conclusions justified by the results? Yes

Is adequate reference made to other work in the field? Yes

## Manuscript Structure

Length of article is: Adequate

Number of tables is: Adequate

Number of figures is: Adequate

Please state any conflict(s) of interest that you have in relation to the review of this paper. None

Interest: Average

Quality: Average

Originality: Average

Overall: Average

Recommendation: Major Revision

## Comments

Prezados autores,

Agradeço a oportunidade de revisar o manuscrito, que aborda um tema de grande relevância. O estudo é bem delineado e contribui para a compreensão do perfil epidemiológico da sífilis gestacional e congênita, bem como da completude das notificações no SINAN.

No entanto, após análise, identifiquei algumas fragilidades metodológicas e estatísticas que comprometem a robustez das inferências. Faço uma análise mais detalhada abaixo dos principais pontos a serem aprimorados. Espero que as sugestões ajudem a fortalecer o estudo.

1. Título e resumo:

O título pode ser mais objetivo e claro com o objeto do estudo, de forma mais concisa: "Perfil epidemiológico e completude das notificações de sífilis gestacional e congênita em uma região de Minas Gerais, 2018-2023".

No resumo, penso que as adequações a serem feitas envolvem os ajustes sugeridos ao longo do parecer. Um foco maior pode ser dado a conclusão, visto que os achados não permitem concluir a segunda parte da sentença.

2. Introdução:

De modo geral, a seção de introdução é coerente e bem estruturada para a proposta apresentada. No entanto, acredito que alguns pontos poderiam ser mais bem articulados, melhorando a clareza e a fluidez do texto. Deixo alguns apontamentos abaixo:

a) Caso seja possível atualizar os dados globais sobre a incidência de sífilis congênita/gestacional e os óbitos, seria interessante para trazer um panorama mais recente. Sugiro manter os valores de 2016 e os atuais, realizando uma comparação entre as estimativas.

b) Na apresentação de informações teóricas, é interessante contextualizar melhor os termos. Por exemplo, na definição de diagnóstico em “tempo oportuno”, seria importante determinar qual o parâmetro considerado (diagnóstico no pré-natal, diagnóstico na fase primária, etc.);

c) A contextualização do cenário local é interessante para melhor direcionamento do estudo para a realidade que será analisada. No entanto, penso que informações sobre a organização da rede, como as regiões de saúde, possam estar no tópico de cenário dos métodos.

d) Também penso que a definição da regional de Ubá, como cenário do estudo, possa estar nos métodos. A justificativa de analisar os dados dessa região se pauta nos próprios valores de taxas de incidência, que superam às médias estaduais e nacionais.

e) Sugiro cautela ao apresentar frases genéricas e truncadas, por exemplo: “a qualidade da assistência pré-natal pode ser questionada”, “continua gerando impactos sociais, econômicos e sanitários”. As ideias precisam ser melhor desenvolvidas, com detalhamento e profundidade.

f) A introdução apresenta bem a problemática, mas poderia fortalecer a argumentação sobre a relevância do estudo. Explicitar de maneira mais clara a lacuna que ele preenche no contexto da vigilância epidemiológica e assistência pré-natal ajudaria a reforçar sua necessidade.

g) O texto menciona que a qualidade do pré-natal pode ser questionada. No entanto, não detalha os fatores específicos que contribuem para essa situação. Vale incorporar informações sobre barreiras ao diagnóstico e tratamento para tornar a argumentação mais robusta.

h) Como um dos focos do estudo é avaliar a completude das notificações, poderia haver uma explicação mais detalhada sobre a importância desse aspecto para a vigilância em saúde e o impacto da incompletude na formulação de políticas públicas.

i) Algumas passagens do texto apresentam informações fragmentadas e desarticuladas, o que pode dificultar a leitura fluida. Sugiro que os autores reformulem algumas frases para que as ideias se conectem de forma mais lógica, ajudando na clareza do material.

j) Minha sugestão de objetivo seria: “descrever o perfil epidemiológico e a completude das notificações de sífilis gestacional e congênita em uma região de alta incidência no estado de Minas Gerais”. Penso que fica mais coerente e adequado com a proposta.

3. Métodos:

O método é coeso e descreve os principais procedimentos adotados para a elaboração do estudo. Alguns pontos podem ser aperfeiçoados para garantir maior clareza e replicabilidade dos passos. Deixo abaixo algumas sugestões para análise e reflexão:

a) A meu ver, o estudo é descritivo e transversal, de caráter descritivo-analítico. É descritivo porque descreve o perfil epidemiológico da sífilis (tabelas 1 e 4) e da completude (tabelas 3 e 5), e transversal porque descreve e compara a prevalência de tratamento adequado (tabela 2).

b) Sugiro reorganizar o método em subseções, para melhor compreensão: Delineamento e contexto; População e critérios de elegibilidade; Fonte de dados e variáveis; Organização e análise dos dados. Verificar o tipo de padronização adotada pela RESS.

c) Seria interessante definir melhor o que foi considerado como duplicidade (mencionando, inclusive, se a base era nominal). Uma gestante pode ter dois registros de sífilis, pois pode ser reinfectada durante a mesma ou em outra gestação. Talvez seja uma limitação do estudo.

d) Como os autores apresentam que houve exclusão das notificações “classificadas pelo sistema como ‘descartado’”, seria interessante apresentar os critérios de elegibilidade para cada um dos casos, de acordo com a padronização mencionada em 2017.

e) Em um certo momento dos métodos os autores mencionam que utilizaram os dados provenientes do TabWin. A ferramenta geralmente usa os bancos em DBF do DATASUS. É importante detalhar como houve o controle das duplicatas na base de dados.

f) O artigo pode melhorar a apresentação dos métodos seguindo o checklist do STROBE (Strengthening the Reporting of Observational Studies in Epidemiology), garantindo transparência e completude na descrição do estudo.

g) Nas variáveis, é importante definir claramente todos os desfechos, exposições, preditores, confundidores em potencial e modificadores de efeito (vide STROBE). Isso inclui, para cada variável, apresentar suas categorias de resposta, assim como foi feito com a dependente.

h) Para cada variável de interesse, forneça a fonte dos dados e os detalhes dos métodos utilizados na avaliação (mensuração: autorrelatada pela pessoa, informada pelo profissional, etc.). Isso é importante para demonstrar a confiabilidade dos dados secundários.

i) A variável “prescrição de tratamento adequada” é fundamental no estudo, mas não está claramente descrita nos métodos. Por exemplo, foi considerada a adesão da gestante ao tratamento, ou apenas a prescrição médica, independente da conclusão?

4. Resultados:

Os resultados são bem estruturados, mas poderiam ser mais interpretativos. Sugiro apresentar os achados em uma sequência mais organizada, evitando repetições e sobreposições de informações. Seguem minhas sugestões para esta seção:

a) Não consegui visualizar a figura suplementar 1. Penso que os autores possam apresentar como elemento textual, evitando suplementos. Algo como: “as taxas de sífilis gestacional e congênita para 2018 foram: XX,X e XX,X; para 2019: XX,X e XX,X” e assim por diante.

b) O terceiro parágrafo introduz o perfil epidemiológico das gestantes. Minha sugestão é evitar a apresentação de variáveis com dado ignorado/em branco, como o caso da escolaridade. Isso porque o estudo já fará uma análise mais acurada das completudes na sequência.

c) Minha sugestão é organizar os resultados da seguinte forma: perfil da sífilis gestacional e congênita (tabelas 1 e 2), prevalência da prescrição adequada ou não (tabela 3), e completude das notificações (tabelas 4 e 5). Ficaria em uma sequência mais lógica e sem redundância.

d) A estratégia adotada pelos autores de apresentar um valor de p para cada subcategoria de uma variável não é a mais adequada. O ideal seria aplicar o teste do qui-quadrado (ou o teste exato de Fisher) comparando todas as categorias da variável simultaneamente.

d.i) Ao comparar separadamente cada subcategoria contra uma referência (por exemplo, branca vs. negra e depois branca vs. amarela), os autores estão realizando múltiplos testes estatísticos, o que pode inflacionar o erro tipo I. Sugiro refazer todos os testes.

d.ii) O qui-quadrado tradicional para tabelas de contingência já testa se há diferença global entre todas as categorias da variável, sem a necessidade de subdividir os grupos inicialmente. A interpretação das variáveis pode considerar os residuais ajustados padronizados.

e) O estudo afirma que a penicilina benzatina foi prescrita para 75,3% das gestantes. No entanto, a taxa de prescrição inadequada foi de 38,8%. O que explica essa discrepância? O problema é a dosagem errada? Falha no seguimento do esquema terapêutico?

5. Discussão:

a) A ferramenta STROBE recomenda que o primeiro parágrafo consista em um resumo dos principais achados relacionando-os aos objetivos do estudo (de forma interpretativa). Sugiro que os autores façam essa adequação, para ser o norte dos argumentos da discussão.

b) A discussão sobre o aumento da sífilis gestacional e congênita é extremamente incipiente. Os argumentos vão além da maior disponibilidade e realização de testagem e do menor uso de preservativos. Existem diferenças no acesso ao diagnóstico e tratamento, incluindo da parceria.

c) Penso que os argumentos precisam avançar mais em termos de hipóteses e implicações. Da maneira apresentada, a discussão está meramente contrastando os resultados com outros estudos nacionais e internacionais, sem agregar valor e argumentação.

d) O diagnóstico no terceiro trimestre não necessariamente implica em diagnóstico tardio. Pode existir o cenário em que a gestante se infecta entre o segundo e o terceiro trimestre e, mesmo testada nos dois primeiros, só tem o diagnóstico no terceiro. Sugiro rever o ponto.

e) Nesse trecho “o diagnóstico de sífilis primária na gestante é raro, sendo mais comum que ocorra na fase latente”, sugiro que substituam por “incomum” ou “menos frequente”, ao invés de raro. Os estudos citados fazem uma hipótese, e não uma afirmação com dados.

f) Penso que a discussão das associações precisará ser revista após a recondução das análises de testes de hipótese. Dessa forma, caso as associações se percam, os autores precisarão adequar os trechos em que falam de associação significativa entre as variáveis.

g) A discussão deve focar nos achados do estudo antes de compará-los com a literatura. Em alguns trechos, há inferências sobre causas que não foram avaliadas. Por exemplo: sugeriu-se que a inadequação do tratamento pode estar relacionada à falta de capacitação profissional.

h) O estudo encontrou associação entre prescrição inadequada do tratamento e variáveis como diagnóstico no terceiro trimestre e ausência de tratamento da parceria sexual, mas a explicação desses achados na discussão poderia ser mais aprofundada.

i) O artigo menciona que a qualidade da informação no SINAN foi inadequada para algumas variáveis essenciais. No entanto, não há uma discussão aprofundada sobre o impacto dessa incompletude nos achados, bem como na vigilância e na assistência em saúde.

j) A discussão menciona a necessidade de capacitação profissional e melhoria da estrutura dos serviços de saúde, mas as sugestões são genéricas. Quais estratégias são recomendadas para melhorar a adesão? Como os serviços podem lidar com a prescrição inadequada?

k) As limitações são citadas, mas poderiam ser mais exploradas. A fase clínica foi ignorada em muitos registros, podendo comprometer a categorização do tratamento. A análise bivariada pode ter encontrado associações espúrias (controladas em uma regressão multivariada).

l) A conclusão resume bem os achados, mas poderia enfatizar com mais clareza as principais recomendações para políticas públicas e ações práticas. A estrutura da discussão deve preparar o leitor para a conclusão, garantindo que não haja elementos novos ou discrepantes.

m) De modo geral, a discussão deve apresentar uma interpretação cautelosa dos resultados (e não apenas unicausal - isto é, com um argumento), considerando os objetivos e as limitações do estudo, os resultados de estudos semelhantes e outras evidências relevantes.

6. Disponibilidade de dados:

Os autores mencionam que os dados estarão disponíveis sob demanda. No entanto, acredito que por terem sido utilizados dados do SINAN, não existe a necessidade de solicitação. A base de dados já está sob domínio público, mesmo que haja algumas divergências com a base local.

7. Referências:

A comparação com a literatura é bem estruturada, mas poderia ser mais criteriosa e atualizada. Algumas referências utilizadas para embasar os achados são relativamente antigas. Se houver publicações mais recentes (<5 anos), seria interessante incorporá-las.

Diante desses aspectos, recomendo revisões substanciais para garantir maior rigor metodológico e clareza na apresentação dos resultados. O parecer acima contém sugestões específicas para aprimorar o manuscrito. Fico à disposição para eventuais esclarecimentos.

